# In Memoriam

**Published:** 1883-02

**Authors:** W. H. Atkinson

**Affiliations:** C. B. A.; New York


### In Memoriam.

At a meeting of the Odontological Society, of Pennsylvania, held January 6, 1883, the following was adopted as a tribute to the memory of a late fellow member:

We desire to express, as far as words can, our deep sorrow for the death of Marshall H. Webb, D. D. S., and our recognition that in his demise we have lost not only a most worthy member, but a sympathizing friend. With deep regret we realize that we shall never again listen to his gentle voice nor feel the force of his personal magnetism.

Death never enters into any family, group of friends, nor an association of any kind, but it trails a shadow to darken some life, to sadden some heart, and render obscure the pathway of the future.

In the death of our colleague and friend we recognize the full force of this, for he not only leaves a sorrowing family but his departure involves a large circle which has felt and will continue to feel its influence far into the future.

Dr. Webb was no ordinary man. He was essentially a leader, not in the sense of arrogant assumption, but as one who took his place through earnest toil, unselfish devotion, and conscientious ambition. He labored that others might grow. He mined that the professional fires might burn all the more brilliantly, and though his day was short, every hour was full, and the record thereof may be filed away in the hearts of his many friends with the indorsement, "Well done, good and faithful servant."

The hour of death is not the hour for critical judgment. Dr. Webb's work belongs to his profession. The ideal that he labored for was his ever-constant inspiration. To accomplish it no sacrifice was too great, no labor too exhausting. No life can but be valued that aims at excellence, but how much more worthy of emulation if the harvest be gathered with the golden grain ripened to perfection! In his special work our friend reached an acme of skill, beyond which he had no superiors. Perhaps he may not have always worked wisely; it may be that his unswerving devotion to his ideal brought him to suffering and death; but, if so, he leaves a monument in his example that will be an honor to his name and a glory to his profession.

We wait, watch, and linger by our dead. Their memory hallows our lives, their deeds are graven on sorrowing hearts; but as we linger the funereal knell reminds us again that change is the law of the Universe, and that in the translation of another we inherit the work of a life, which should be an ever present incentive abiding with us forever.

*Resolved*—That a copy of the foregoing be transmitted to the family of the deceased with the assurance of our deep sympathy with them in their affliction, and also forwarded to the dental journals for publication.

DR. TAFT; *Dear Sir*:—Dr. Marshall Hickman Webb died January 1, 1883, at his home in Lancaster, Pa., at 5 A. M., of cancer of the colon, from which he had suffered two years. He was born at Marlsborough, Chester County, Pa., Oct. 28, 1844, making him 38 years old. He was graduated in 1867 by the Philadelphia Dental College, and practiced in Lancaster ever since. Attended the Congress at London and demonstrated to our English brethren American superiority in operating. During his illness he suffered greatly, but while in bed wrote "Notes on Operative Dentistry," which is in press and shortly to be published by the S. S. W. Co. He was lecturer on operative dentistry and histology at the University of Pennsylvania, and member of the Harris Dental Association of Lancaster, County, the State Dental Association of Pennsylvania, and the American Dental Association. Yours Hastily and Faithfully,

W. H. ATKINSON, C. B. A.

New York, January 19, 1883.

